# Effective medical leadership in times of emergency: a perspective

**DOI:** 10.1186/s40696-016-0013-8

**Published:** 2016-02-06

**Authors:** Oded Hershkovich, David Gilad, Eyal Zimlichman, Yitshak Kreiss

**Affiliations:** 1 Medical Corps, Israeli Defense Forces, Tel Hashomer, Israel; 2 Chaim Sheba Medical Center, Tel Ha Shomer, Israel; 3 Department of Military Medicine, Hebrew University, Jerusalem, Israel

## Abstract

Leadership, and more specifically medical leadership, is an unmeasured potential that has the power to influence every aspect of a person’s professional life and its challenges and is more evident in times of emergency. Medical leadership is receiving increasing recognition especially in discussing actions to be taken in times of stress and emergency. We propose a comprehensive conceptual model that examines the elements that build successful medical leadership, especially during emergency scenarios. The model is based on two sets of medical leadership capabilities and skills, while the first set is more relevant to everyday challenges, the second set represents abilities and characteristics that arise mostly during emergencies. The model gathers together the characteristics and abilities of the medical leader based on our unique personal experiences during conflicts, terror, civilian challenges and numerous humanitarian missions. This article suggests a framework for the foundations on which the medical leader’s education should be built and describes our perception of how to establish medical leadership, its unique elements and the processes leading to outstanding performance in times of emergency.

## Background

Being at the center of clinical service delivery, physicians are the ideal leaders for health care in the twenty-first century [1]. As medical education evolves with the passage of time and adapts itself to an ever-increasing knowledge base, the area of physician leadership remains underdeveloped [2]. Physicians are trained in the diagnostic, therapeutic and administrative aspects of patient care, but not in the theoretical and practical aspects of assuming and delivering leadership. The medical curricula in many medical schools fail to incorporate an organized leadership training (designated courses or practice) as part of both graduate and post-graduate studies and this stems from a lack of recognition of the critical role physicians must play as leaders. Nowhere is the lack of a leadership role more evident than in times of emergency.

Effective administration of healthcare in an emergency setting is considerably more complex than the regular patient–doctor interactions characteristic of routine visits due to the complexity and uncertainty typical of such an environment. In such situations, order must be established, needs assessed and resources allocated effectively, and therefore the affected public seeks leadership in those who are perceived to be at the center of clinical service delivery—the physicians.

While medicine focuses on decision making at the individual physician–patient level, leadership involves stepping back and examining problems at a higher level thus requiring the ability to view issues broadly and systemically [3]. Leadership in emergency situations demands recognition of needs, organized learning and practice of more than just basic skills. The person who assumes the role of leader in this setting must be able to rapidly analyze a complex environment, assess where and what sort of help is required, assemble an effective, multidisciplinary staff of care providers, and communicate effectively among the staff and the population in need. Such a leader needs to make informed decisions rapidly, and be capable of ongoing self-assessment and adaptation to unfamiliar and rapidly changing conditions, thus, assuring the seamless provision of optimal care.

This article aims to present a comprehensive conceptual model based both on theoretical knowledge and our practical leadership experience in the Israeli Defense Forces (IDF) Medical Corps with regards local emergencies, numerous humanitarian efforts and disaster management around the world [4–8].

We describe the model, with the particular skills necessary for physician leaders to learn, as a key to achieving effective performance in times of need and also as a platform for a systematic medical leadership development process.

## The model

The suggested model is integrative and arranged around two sets of skills: The first are the basic leadership skills—those required for medical leadership mostly during quiet times and that may be adapted to emergency situations. The second are emergency applicable leadership skills which outline the additional, unique set of skills mostly required specifically for emergency scenarios.

### Basic leadership skills

Basic leadership skills include: operating with clear vision and organizational values, implementing strategic and tactical planning and developing communication, negotiation and collaboration skills.

*Operating with clear vision and organizational values* is important for every leader in any situation. Since the primary focus of physicians is on their professionalism and practice, medical leadership requires modifying this mind set to more strategic thinking, consolidating organizational thought, developing a vision, aligning viewpoints, and setting goals. It is vitally important to educate medical professionals to understand they have a responsibility to contribute to the effective operation of the organization in which they work and to its future direction and objectives.

The importance of those skills increases intensely during emergency scenarios when uncertainty and time pressure emphasize the need for an almost automatic response. Leaders are expected to keep mission and organization values in mind in the decision-making process and stay true to their values even when it may be tempting to back out [9]. A clear vision based on well-established values guides leaders and individuals to act properly, sometimes against instincts or even under life threatening situations, like in extreme disasters such as the earthquake in Haiti [5], the 9/11 disaster in New York City [10] or the genocide in Rwanda [11].

#### Strategic planning is a necessity for medical leadership

Unfortunately, young physicians, who focus on a daily, tactical level that is primarily one-on-one patient–physician interaction, come out of medical school with an inadequate ability to implement strategic thinking. Gruthrie et al. [12] claims that doctors are trained to be individual performers and lack organizational capabilities. During emergency situations, implementing strategic planning is essential. Emergency situations are usually characterized by highly demanding surroundings with multiple constraints mandating effective resource allocation, especially in the early response phase.

The IDF’s experience in disaster management [5, 13] supports the crucial role of long term planning as a key factor for preparedness, readiness, quick response and later-effective decision making. This planning process should be *continuous*—beginning before the mission, *adaptable*-to the analysis of the specific situation, and *integrative*—based on manpower, equipment, supply and operational organization.

However, strategic level of planning alone is not enough in times of emergency. The medical leader should also be able to create short term, adaptable and flexible planning at the tactical level which includes role rotating of medical personnel, flexibility in team structure, and creative solutions. Moreover, due to the unpredictability of the situation, physician leaders should be able to shift from the strategic level of planning to the tactical level and switch between them according to the changing environment, the needs and the required adjustments [5, 14].

#### Communication, negotiation and collaboration as a strategy

Leadership is not only about the leader himself; it is also about developing the personal qualities of communication and negotiation to work effectively with others. Physician leaders should become the mediators between their team and the organizational management, minimizing miscommunication and maximizing agreement and understanding.

These capabilities may be developed gradually in times of quiet, but should be practiced intensely and adopted more quickly in emergency settings which demand quick team building, managing heterogeneous staff, dissolving conflicts between team members and building group resilience.

In crisis situations, working alone is simply not an option, so collaboration is vital. This task may sometimes be complicated in an unfamiliar environment with potential barriers impeding the collaboration such as language, political and legal issues, differences in accepted working procedures and lack of time to create firm interpersonal connections. Therefore, the skills of communication and negotiation should be adapted and developed also towards communicating with new partners and building the outer circles of cooperation. Shirley and Mandersloot [15] describe their experience in the intensive care unit of the Royal London Hospital after the first suicide bombing in London in Jul 2005. They claim that the successful outcome of the vast majority of patients was multi-factorial and may be attributed, in part, to the cooperation between designated receiving hospitals, neighboring hospitals, ambulances, police, and other rescue services, thus ensuring a structured and coordinated response.

Our strategy during emergency situations is to encourage operating a variety of communication channels and negotiating with local operators, international organizations, the local population and other medical teams. We educate our medical leaders to adopt collaboration as a strategy and to understand they are obligated to overcome barriers of language and culture and to find the ways to bridge differences in working procedures and legalities.

### Emergency applicable leadership skills

Performing in emergency situations is a higher level of complexity, exposing the medical leader to a different, convoluted, irregular operating arena which requires the ability to process and analyze a new environment, quick decision-making capabilities, and effective management of people, all under emergency conditions.

*The ability to process and analyze a complex environment* in an orderly manner is an essential element of effective leadership during medical emergencies. This environment, which is influenced by safety considerations and a high level of uncertainty, requires the leader to process information in a clearly defined, organized manner, in order to analyze several operational alternatives in a vastly reduced window of reaction time. Moreover, in emergency situations the medical outcome may be influenced by different non-medical parameters, so we educate and train our medical leaders to broaden their perspective and to add to their emergency analysis other aspects such as social, legal, political and cultural considerations.

Hannah et al. [16] suggest that the ability to give meaning and to simplify complex situations to formed schemas for individuals are critical intervention points for leadership. The leader’s judgment and action (or inaction) will determine the potential influence of an emergency situation on the outcome.

*Quick decision*-*making based on situational analysis, knowledge and experience* is expected of medical leaders in extremely difficult situations. Our experience suggests that a medical leader who is not qualified enough would not make the correct decisions under pressure or would be too slow to react on time. Different types of decisions are required during emergency management both at the leader level and at the individual level. Many of them, like hospitalization dynamics, work load of professional personnel, and level of security, have the potential to threaten the accomplishment of the mission, but probably the most complicated for the medical leader in emergency scenarios are the unusual ethical dilemmas.

Emergencies and disasters posit personal, professional, and public interests one against the other and create complex ethical dilemmas which are at the center of emergency management. These dilemmas include treatment priorities, triage considerations, dealing with surge capacity and resource allocation according to survival chances and not according to the individual needs. Medical leaders must be prepared to confront these complex ethical issues in order to be able to make decisions and take relevant action [13]. We encourage medical leaders to use generic tools during emergencies, but also to create on-site leadership supporting mechanisms, adjusted to the specific environment, that will help establish an ethical and practical system of medical priorities in a chaotic setting. Such a structured system of decision making was described by us [7] and similar processes were reported by others after the strike of Hurricane Katrina in 2005 and after Hurricane Sandy in 2012 [17–19].

#### Managing people and building their endurance

A major role for the leader, is to continuously strengthen the team endurance before the mission, and more importantly, during the mission. Building this kind of endurance in a disorganized setting requires leaders to take-care of their teams, to understand their needs even before they are expressed, and to identify desires, ambitions, tensions and fears in order to steer them effectively towards the mission. This can be challenging for medical leaders because their team may be laboring under a heavy work load, exposed to mental and physical stress, or confronting significant risks. To deal with these challenges Hannah et al. [16] suggest that leadership during actual acute events should be more directive and transactional. Leaders have to understand the milieu of their team and their organizational behavior and work continuously to motivate them to build and strengthen their commitment to the mission.

When trying to strengthen team cohesion and minimize the mental burden during emergencies situations, medical leaders should address several aspects:

*The anti*-*chaos effect* The best way to manage and motivate people working in a disorganized environment or chaos is to create order. Medical leaders should enhance transformation of individual professionals into a team with strong cohesion using ceremonies and symbols, operating under a strict schedule and organizational discipline, and establishing order when needed [5, 19].

*The “big picture” effect* An important dilemma of leading people in emergency situation is the question of how much information should be shared with the personnel. Sharing information and exposing people to the general picture may help them cope better with the stress, but sometimes it may cause frustration and increase the mental burden. The IDF experience proves it is better to share the “big picture” in most cases, but to continuously work to identify those groups and individuals who need a different or modified approach.

*Rest and debriefing effect* When faced with people who are in danger and need of assistance, medical personnel in emergency situations tend to work themselves around the clock and quickly wear themselves out. This can lead people to work less effectively with time and may endanger their mental health. The leader should sometimes work against this tendency and force his staff to rest and re-charge. Another important task of the leader is to establish mechanisms for mental debriefing on a regular time schedule in order to provide them with the opportunity to relieve stress. This task should continue even after the mission ends, due to late onset of reactions to such scenarios among medical personnel [20].

## Conclusion

Leadership is one of the most researched social phenomena, with a wide spectrum of definitions, parameters and theories. Certain parameters discussed in our model are generic and might apply to leadership in emergency situation not specifically medical ones; nevertheless, medical leadership has its own uniqueness and complexity as discussed above.

Regardless of their background, education, training, environment and skills, each leader will use a different leadership style by finding a unique element within themselves, embracing it and using it. It should be acknowledged that leadership style depends not only on one’s personality, but also on the environment one is operating in and on the team members involved [21]. No matter which model is used, the most important factor for effective leadership is the interface between personality, skills and capabilities, the interaction with those being led and influencing the immediate surrounding environment. Effective leaders should possess the ability to switch between leadership styles suited to the different situations and adjust them accordingly [22].

Effective crisis leadership lies also on the understanding that the inflow of knowledge is so extensive that one person, cannot contain the entire scope of information [23, 24], and therefore, as Pearce suggests, leadership is actually an outcome of a web interaction of groups rather than just a direct influence of a leader [25, 26]. This dynamic leadership evolves while different proxies interact inside this web and create new types of behaviors and actions making those organizations more creative, flexible, adaptive and affective to deal with the uncertainties. The leadership role, accordingly, is to create those opportunities for creativity and innovativeness.

Our suggested model incorporates the required skills and capabilities developed in times of quiet in preparedness for times of emergency. Assuming that effective leadership requires basic personal capabilities and motivation in order to lead and influence others, one should bear in mind that it can be built and developed just as any other skill [27]. Moreover, while many physicians possess character traits essential to leadership, and may have acquired relevant leadership skills in the course of their lives and careers, the medical establishment neglects to incorporate formal leadership training as an essential component of every physician’s education, at its own—and society’s—peril.

Therefore, accepting a comprehensive conceptual model to be used as the core for education, training and preparedness is of great importance. The suggested model is incorporated in the medical education curricula of the “Tsameret” Program of Excellence for military physicians in the IDF Medical Corps and we propose it as a generic educational concept for developing medical leadership.
